# Location-specific outcomes and complications of endoscopic transorbital approaches: A systematic review with novel anatomical grouping

**DOI:** 10.1016/j.bas.2025.105895

**Published:** 2025-12-03

**Authors:** Sophia C. Lam, Jason Y.S. Cheung, Ben C.F. Ng, Hunter K.L. Yuen, Calvin H.K. Mak

**Affiliations:** a Department of Neurosurgery, Queen Elizabeth Hospital, Hong Kong, China; b Faculty of Medicine, The Chinese University of Hong Kong, Hong Kong, China; c Department of Ophthalmology and Visual Sciences, The Chinese University of Hong Kong, Hong Kong, China

**Keywords:** Endoscopic surgery, Transorbital approach, ETOA

## Abstract

**Introduction:**

Endoscopic transorbital approach (ETOA) is gaining recognition due to lower complication rates and better cosmetic outcomes. Nonetheless, there is no clear anatomical grouping system for lesions that ETOA can address, and location-specific complication rates are still lacking.

**Research question:**

This systematic review provides an anatomical grouping system for ETOA and analyse the location-specific surgical risks and outcomes.

**Material and methods:**

Based on the PRISMA guideline, articles with keywords “Endoscopic” and “Transorbital” were searched and analysed. The cases included are regrouped based on four anatomical locations (I - orbital, II - cavernous sinus, III - extradural, IV - intradural), and outcomes are studied respectively.

**Results:**

Data from 28 published articles with 382 patients were identified. There were 113 orbital lesions, 58 cavernous lesions, 18 extradural lesions, and 150 intradural lesions. There was significant post-operative visual acuity improvement in Groups I (70.6 %), II (56.3 %), and IV (63.3 %). Proptosis shows notable improvement rates across all groups, particularly in Groups II (95.7 %) and IV (87.0 %). There was an observed difference in the rate of CSF leak depending on the location of the lesion: 0 % in both Group I and II versus 11.8 % in Group III and 3.4 % in Group IV (*p=0.005)*.

**Discussion and conclusion:**

This systematic review proposed an anatomical grouping system to analyse location-specific outcomes for ETOA. Our findings highlighted the significance of this new classification for anatomy-based risk assessment. Future, larger-scale, and multicenter research will generate more data, allowing for further stratification of outcomes based on specific pathology subtypes.

## Highlights

• A proposed anatomical grouping system aims to analyse location-specific surgical risks and outcomes for ETOA.
• Significant differences in surgical outcomes were identified based on the anatomical location of the lesion.
• The results once again highlighted the efficacy of ETOA.

## 1 Introduction

The endoscopic transorbital approach (ETOA) provides a new corridor in skull base surgery, allowing access to orbital and intracranial pathologies. This minimally invasive approach is gaining recognition as it provides lower complication rates and better cosmetic outcomes as compared to the traditional transcranial approach. There has been a booming number of publications and case series on ETOA, demonstrating its safety and efficacy for a wide range of pathologies in different anatomical locations. Examples of these pathologies range from orbital cavernous haemangiomas to sphenoid wing meningiomas and intra-axial gliomas. Besides excision of lesions, ETOA has also been proven helpful in trauma scenarios, such as orbital decompression and repair of cerebrospinal fluid (CSF) leaks.

To date, there is no widely adopted anatomical grouping system for lesions that ETOA can address. Mathios et al. previously described seven “target zones” as an anatomical grouping(Mathios et al., 2023), yet data on stratified outcomes and complications remain lacking. With the increasing adoption of ETOA for clinical cases, there is a need for a grouping system that can provide data for location-specific outcomes and complications of ETOA to provide evidence that skull base surgeons can base their clinical decisions on. Hence, this systematic review aims to provide a clinically relevant anatomical grouping system for ETOA, based on available published data.

## 2 Methods

In accordance with the Preferred Reporting Items for Systematic Reviews and Meta-Analyses (PRISMA) guidelines(Moher et al., 2015), two independent investigators conducted systematic literature searches of the online database PubMed/MEDLINE, among articles published between January 2010 and March 2025. The combination of keywords (“Endoscopic” AND “Transorbital”) was applied as our search string, and the relevant articles retrieved by the reviewers were compared and compiled.

### 2.1 Inclusion criteria

Inclusion criteria for the study were: 1) Any articles providing a report of ≥5 cases of patients who underwent endoscopic surgery via the transorbital approach, and 2) Studies satisfying the previous criteria that concomitantly employed other endoscopic or open approaches (e.g., endoscopic endonasal). Only articles with full text published in the English language were included.

### 2.2 Exclusion criteria

The exclusion criteria were: 1) Studies using the endoscopic approach not via the transorbital route, 2) Studies using the transorbital approach but not involving endoscope, 3) Pure anatomical studies, 4) Case reports with case number <5 patients, 5) Case series in which patient data were presented as a summary but not discussed individually, and 5) Reviews, letters, and other unrelated publications.

### 2.3 Data extraction and synthesis

Information that was collected from the eligible articles included patient demographics, pathology, extent of excision (if applicable), surgical approach, follow-up duration, presenting symptoms/signs (pre-operative visual acuity, visual field, extraocular movement, proptosis), surgical outcomes (post-operative visual acuity, visual field, extraocular movement, proptosis), and complications (CSF leak, ptosis, recurrence, etc). Note that visual acuity and visual field outcomes are reported separately whenever specified by the study; if only “vision” was reported by the original case series, the outcomes were classified under visual acuity in our review.

Based on the location of pathology, patients were classified into four groups for separate evaluation: Group I – Orbital, Group II – Cavernous sinus, Group III – Extradural, and Group IV – Intradural. Classification was based on the following criteria:

1. Any pathology that requires incision of the meningeal dura during operation (e.g., meningiomas, temporal lobe lesions) should always be classified as intradural (Group IV), regardless of any orbital invasion.
2. A cavernous sinus (CS) lesion (Group II) is not necessarily intradural, although it may certainly be (e.g., CS meningiomas). Any pathology that involves the CS or its walls (e.g., trigeminal schwannoma) may be classified as cavernous.
3. An extradural lesion that is partly or totally within the orbit should be classified as orbital (Group I).
4. If a lesion cannot be classified as orbital (Group I), cavernous (Group II), or intradural (Group IV), it may then be considered extradural (Group III).
5. If a lesion spans multiple locations, it should be classified according to its site of origin (epicenter). E.g., trigeminal schwannomas extending into the orbital apex are classified as cavernous (Group II) instead of orbital (Group I).

### 2.4 Risk-of-bias assessment

The methodological quality of included case series was assessed using the Joanna Briggs Institute (JBI) Critical Appraisal Checklist for Case Series. Two independent reviewers applied the checklist to each included study, rating each item as “Yes,” “No,” “Unclear,” or “Not Applicable.” Disagreements were resolved through discussion. Studies were not excluded based on quality scores, but the results were used to describe the risk of bias and inform the interpretation of findings.

## 3 Results

**Fig. 1:**
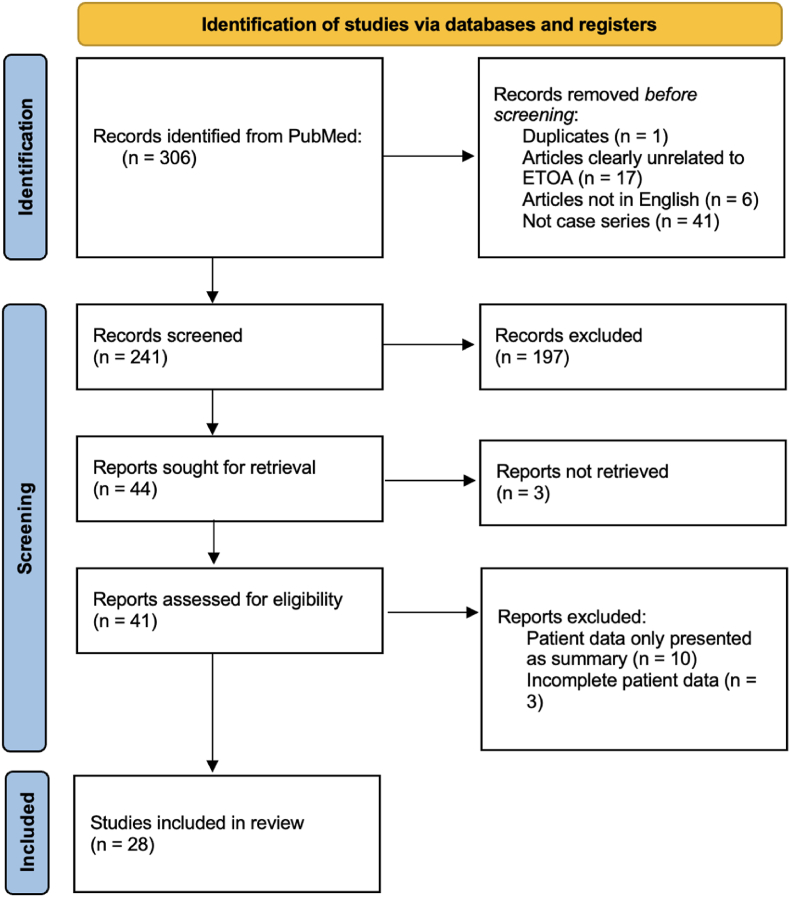
PRISMA flowchart for selection of studies included in this review.

A total of 306 articles were identified and 28 articles were included after screening (see Table 1) based on our search criteria (PRISMA flowchart shown in Fig. 1). The mean JBI critical appraisal score is 7.8 out of 10, reflecting good methodological quality and a low to moderate overall risk of bias within the limitations inherent to retrospective and prospective case series designs. From the included studies, a total of 382 patients were identified. These patients are grouped based on the anatomical locations of their pathologies. The results are as follows (Summarized in Table 2):

**Table 1:** Selected papers for the present review. ∗This score reports the number of items in the checklist rated as “Yes”, out of 10 questions.

Author	Year	Nature of paper	Retrospective/prospective?	Level of evidence	JBI checklist score∗
Moe et al. (Moe et al., 2010a)	2010	Case series	Prospective	IV	4/10
Moe et al. (Moe et al., 2010b)	2011		Prospective		8/10
Lim et al. (Lim et al., 2012)	2012		Prospective		8/10
Dallan et al. (Dallan et al., 2016)	2016		Retrospective		7/10
Dallan et al. (Dallan et al., 2017)	2018		Retrospective		7/10
Kong et al. (Kong et al., 2019)	2018		Prospective		6/10
Jeon et al. (Jeon et al., 2019)	2018		Prospective		8/10
Miller et al. (Miller et al., 2019)	2019		Retrospective		8/10
Park et al. (Park et al., 2020a)	2020		Retrospective		8/10
Goncalves & Lubbe (Goncalves and Lubbe, 2020)	2020		Retrospective		9/10
Park et al. (Park et al., 2020b)	2020		Retrospective		9/10
Jeon et al. (Jeon et al., 2020)	2020		Retrospective		8/10
Woo et al. (Woo et al., 2020)	2021		Retrospective		9/10
Noiphithak et al. (Noiphithak et al., 2020)	2021		Retrospective		8/10
Lee et al. (Lee et al., 2021)	2021		Retrospective		7/10
Park et al. (Park et al., 2021)	2021		Retrospective		9/10
Lim et al. (Lim et al., 2022)	2022		Retrospective		8/10
Han et al. (Han et al., 2023)	2023		Retrospective		9/10
Kim et al. (Kim et al., 2024)	2023		Retrospective		7/10
Di Somma et al. (Di Somma et al., 2023a)	2023	Case series and literature review	Retrospective		7/10
Mathios et al. (Mathios et al., 2023)	2023	Case series	Retrospective		9/10
Carnevale et al. (Carnevale et al., 2023)	2024		Retrospective		8/10
Zoia et al. (Zoia et al., 2024a)	2024		Retrospective		8/10
Di Somma et al. (Di Somma et al., 2023b)	2024		Retrospective		8/10
Jeon et al. (Jeon et al., 2024a)	2024		Retrospective		8/10
Zoia et al. (Zoia et al., 2024b)	2024		Retrospective		8/10
Ricciuti et al. (Ricciuti et al., 2024)	2024		Retrospective		8/10
Jeon et al. (Jeon et al., 2024b)	2024		Retrospective		8/10

**Table 2:** Location-specific post-operative outcome and complication rates.

	Group I: Orbital (n = 113)	Group II: Cavernous (n = 58)	Group III: Extradural (n = 18)	Group IV: Intradural (n = 150)	All (n = 339)	*Chi-squaredtest*
**Post-operative improvements**						
Visual acuity	70.6 % (12/17)	56.3 % (9/16)	0 % (0/1)	63.3 % (31/49)	62.7 % (52/83)	*p = 0.489*
Visual field	0 % (0/1)	100 % (9/9)	n/a	40 % (2/5)	73.3 % (11/15)	***p = 0.012***
EOM deficit/Diplopia	9.1 % (1/11)	55.6 % (5/9)	100 % (1/1)	62.5 % (10/16)	45.9 % (17/37)	***p = 0.026***
Proptosis	32.1 % (17/53)	95.7 % (22/23)	75 % (3/4)	87.0 % (60/69)	68.5 % (102/149)	***p < 0.001***
**Complications**						
CSF leak	0 % (0/111)	0 % (0/54)	11.8 % (2/17)	3.4 % (4/116)	2.0 % (6/298)	***p = 0.005***
EOM deficit/Diplopia	8.0 % (9/112)	9.6 % (5/52)	0 % (0/17)	6.8 % (8/118)	7.4 % (22/299)	*p = 0.599*
Ptosis	8.2 % (9/110)	20.5 % (9/44)	0 % (0/15)	6.8 % (6/88)	9.3 % (24/257)	***p = 0.032***

### 3.1 Group I: orbital lesions

Of the 382 patients, 113 (29.6 %) had orbital lesions. The mean age was 52.2 (±16.3). There were 54 males and 59 females. Pathologies included meningioma (n = 20), schwannoma (n = 10), cavernous haemangioma (n = 10), orbital abscess (n = 11), and other pathologies (n = 62) including metastatic carcinoma, mucocele, dermoid cysts, et cetera. Gross total resection (GTR) rate was 50.4 %, subtotal resection (STR) rate was 13.3 %, and biopsy rate was 13.3 %. Among the cases with available data, the mean follow-up duration was 28.4 ± 23.8 months, and the recurrence rate was 8.9 %.

**Visual acuity**: 39 cases have documented pre-operative VA status, with 17 (43.6 %) of them reported to have pre-operative VA impairment. 12 out of 17 patients (70.6 %) reported VA improvement post-operatively. No patients have reported new VA impairment post-operatively.

**Visual field:** 29 cases have documented pre-operative VF status, with only 1 (3.44 %) reported to have pre-operative VF impairment. The patient did not show VF improvement post-operatively.

**Proptosis**: 86 cases have documented pre-operative proptosis status, with 53 (61.6 %) of them reported to have pre-operative proptosis. 17 out of 53 patients (32.1 %) reported improvement in proptosis post-operatively.

**Diplopia or EOM limitation**: 27 cases have documented pre-operative diplopia or EOM status, with 11 (40.7 %) of them reported to have pre-operative diplopia or EOM limitation. 1 out of 11 patients (9.1 %) reported diplopia or EOM improvement post-operatively. Out of 112 reported cases, 9 patients (8.0 %) have reported new diplopia or EOM limitation post-operatively, with 8 cases (88.9 %) being a transient deficit.

**CSF leak**: No post-operative CSF leak was reported among the available data of 111 patients with orbital lesions.

**Ptosis**: 9 patients (8.2 %) developed new ptosis post-operatively among the 110 available patients, with 7 cases (77.8 %) being transient.

### 3.2 Group II: cavernous sinus lesions

58 out of the 382 patients (15.2 %) had cavernous sinus lesions. The mean age was 51.3 (±12.8). There were 16 males and 42 females. Pathologies included meningioma (n = 31), schwannoma (n = 21), cavernous haemangioma (n = 10), and other pathologies (n = 6) including abscess, prolactinoma, chondrosarcoma, et cetera. GTR rate was 48.3 %, STR rate was 39.7 %, and biopsy rate was 3.4 %. Among the cases with available data, the mean follow-up duration was 15.0 ± 11.3 months. No data on the recurrence rate were available for this group of patients.

**Visual acuity**: 44 cases have documented pre-operative VA status, with 16 (36.4 %) of them reported to have pre-operative VA impairment. 9 out of 16 patients (56.3 %) reported VA improvement post-operatively.

**Visual field:** 41 cases have documented pre-operative VF status, with 9 (22.0 %) of them reported to have pre-operative VF impairment. All patients (100 %) reported VF improvement post-operatively.

**Proptosis**: 47 cases have documented pre-operative proptosis status, with 23 (48.9 %) of them reported to have pre-operative proptosis. 22 out of 23 patients (95.7 %) reported improvement in proptosis post-operatively.

**Diplopia or EOM limitation**: 41 cases have documented pre-operative diplopia or EOM status, with 9 (22.0 %) of them reported to have pre-operative diplopia or EOM limitation. 5 out of 9 patients (55.6 %) reported diplopia or EOM improvement post-operatively. Out of 52 reported cases, 5 (9.6 %) have reported new diplopia or EOM limitation post-operatively, with all being transient deficits.

**CSF leak**: No post-operative CSF leak was reported among the available data of 54 patients with cavernous sinus lesions.

**Ptosis**: 9 patients (20.5 %) had new ptosis post-operatively among the available data of 44 patients, which all are transient.

### 3.3 Group III: extradural lesions

18 out of the 382 patients (4.7 %) had extradural lesions. The mean age was 42.7 (±15.3). There were 11 males and 7 females. Pathologies mainly included mucocele/mucopyocele (n = 10), with other pathologies (n = 8) including abscess, chordoma, plasmacytoma, epidermoid cyst, et cetera. The extent of resection is not applicable for mucocele, mucopyocele, and abscess. For the remaining pathologies, the GTR rate is 85.7 % and the STR rate is 14.3 %. Among the cases with available data, the mean follow-up duration was 19.0 ± 17.8 months. There was inadequate reporting of the recurrence rate, and therefore, the data generated is not representative of this group.

**Visual acuity**: 4 cases have documented pre-operative VA status, with 1 (25 %) of them reported to have pre-operative VA impairment. No patients reported VA improvement post-operatively.

**Visual field:** 3 cases have documented pre-operative VF status, none of which have pre-operative VF impairment.

**Proptosis**: 5 cases have documented pre-operative proptosis status, with 4 (80 %) of them reported to have pre-operative proptosis. 3 out of 4 patients (75 %) reported proptosis improvement post-operatively.

**Diplopia or EOM limitation**: 3 cases have documented pre-operative diplopia or EOM status, with 1 (33.3 %) of them reporting pre-operative diplopia or EOM limitation. This patient reported diplopia or EOM improvement post-operatively. None of the patients reported new diplopia or EOM limitation post-operatively.

**CSF leak**: 2 cases (11.8 %) of post-operative CSF leak were reported among the available data of 17 patients with extradural lesions.

**Ptosis**: No patients developed new ptosis post-operatively among the available data of 17 patients.

**Other complications**: 2 cases (11.8 %) out of the 17 reported cases had other minor complications, which were V1 paresthesia and periorbital edema, respectively.

### 3.4 Group IV: intradural lesions

Of the 382 patients, 150 (39.3 %) had intradural lesions. The mean age was 53.2 (±16.3). There were 32 males and 90 females. Pathologies mainly included meningioma (n = 120), glioma (n = 19), metastatic brain tumour (n = 6), and other pathologies (n = 5) including cavernous malformation, arachnoid cyst, and choroid plexus papilloma. GTR rate was 58.7 %, STR rate was 32.0 %, biopsy rate was 0.7 %, and the resection date was not available for the remaining 8.7 % cases. Among the cases with available data, the mean follow-up duration was 24.5 ± 20.3 months. There was inadequate reporting of the recurrence rate, and therefore, the data generated is not representative of this group.

**Visual acuity**: 109 cases have documented pre-operative VA status, with 49 (45.0 %) of them reported to have pre-operative VA impairment. 31 patients (63.3 %) reported VA improvement post-operatively.

**Visual field:** 72 cases have documented pre-operative VF status, with 5 (6.94 %) of them reported to have pre-operative VF impairment. 2 patients (40 %) reported VF improvement post-operatively.

**Proptosis**: 104 cases have documented pre-operative proptosis status, with 69 (66.3 %) of them reported to have pre-operative proptosis. 60 out of 69 patients (87.0 %) reported proptosis improvement post-operatively.

**Diplopia or EOM limitation**: 62 cases have documented pre-operative diplopia or EOM status, with 16 (25.8 %) of them reported to have pre-operative diplopia or EOM limitation. 10 (62.5 %) patients reported diplopia or EOM improvement post-operatively. From available data, 8 out of 118 patients (6.8 %) reported new diplopia or EOM limitation post-operatively.

**CSF leak**: 4 cases (3.4 %) of post-operative CSF leak were reported among the available data of 116 patients with extradural lesions.

**Ptosis**: 6 patients (6.8 %) developed new ptosis post-operatively among the 88 available patients, all of which were transient ptosis.

**Other complications**: 29 cases (23.4 %) out of the 124 reported cases had other complications, which are mainly ipsilateral V1 or V1 paresthesia (n = 12), periorbital edema (n = 8), intraocular haemorrhage (n = 2), haematoma (n = 2), eyelid necrosis or ectropion (n = 2), keratopathy (n = 1), pseudomeningocele (n = 1), and visual field defect (n = 1).

## 4 Discussion

Since Moe et al. first described ETOA in 2010(Moe et al., 2010a), its application has since been expanded significantly to encompass a diverse array of orbital and skull base pathologies including meningiomas, trigeminal schwannomas, orbital tumors, intra-axial lesions, and others (Goncalves and Lubbe, 2020; Park et al., 2020b, 2021; Zoia et al., 2024b). Yet, the heterogeneity of these pathologies coupled with their distinct anatomical locations has marked influence on resection rates, complication profiles, and functional outcomes. Consequently, aggregate analyses of ETOA outcomes in the absence of anatomical stratification may obscure the true efficacy and safety of this approach across specific clinical contexts.

To the best of our knowledge, this systematic review represents the first effort to categorise ETOA cases based on four distinct anatomical locations of the underlying pathology, and analyse their respective surgical outcomes and major complications to aid clinical adaptation. These anatomical locations involve different neurovascular structures and technical challenges, thus warranting the stratification of outcomes by location and allowing the identification of anatomical-specific risks that should be considered when offering ETOA.

Our study again demonstrated the safety and efficacy of ETOA as a minimally invasive surgical approach for skull base and orbital lesions while highlighting its varied efficacy at approaching distinct anatomical compartments. Although our statistics remains exploratory in view of data heterogeneity, our results demonstrated that the rate of postoperative CSF leak has statistically significant differences (*p = 0.005*) depending on the location of the lesion: 0 % in both Group I (orbital) and Group II (cavernous) versus 11.8 % and 3.4 % in Group III (extradural) and Group IV (intradural) lesions respectively. While prior studies have not explicitly analysed CSF leak risks by anatomical location, recent reviews report CSF leak rates for ETOA in meningioma cases, including spheno-orbital meningiomas, ranging from 2 % to 5 % (Di Somma et al., 2023a; Vural et al., 2021; Corvino et al., 2022, 2025; Agosti et al., 2023; Ribeiro et al., 2025), which aligns closely with our findings for Group IV (intradural) lesions. Similarly, Corvino et al. reported a 0 % CSF leak rate across 60 cases of trigeminal schwannomas treated with ETOA (Corvino et al., 2025), consistent with our results for Group II (cavernous) lesions. These concordant findings suggest the validity of our anatomical classification in capturing anatomical-specific risk profiles.

We demonstrated the statistical significantly higher rate (*p = 0.032*) of transient post-operative ptosis identified in Group II lesions (20.5 %) compared to other groups (0 %–8.2 %). This elevated incidence may be explained by involvement of the oculomotor nerve, adding a neurogenic component to the development of ptosis, in addition to direct damage of the levator aponeurosis during interfascial dissection to access the superior orbital rim (Moe et al., 2010a). De Simone et al. also noted an increased incidence of ptosis in trigeminal schwannoma cases treated with ETOA compared with microsurgical excision (De Simone et al., 2024b). This finding, combined with ours, warrants further improvement of the surgical techniques in Group II (cavernous) patients to prevent and reduce the risk of post-operative ptosis.

Regarding the resolution of common pre-operative morbidities, we found that the rate of proptosis and visual improvements were highest among Group II (cavernous, 95.7 %) and Group I (orbital, 70.6 %) lesions respectively. For Group IV (intradural) lesions, visual acuity improvement was observed in 63.3 % of cases, and proptosis improvement in 87.0 %, roughly aligning with reported ranges in the literature of 66.7–93 % for visual acuity and 79.4–100 % for proptosis improvement (Di Somma et al., 2023a; Corvino et al., 2022; Agosti et al., 2023). These findings may reflect distinct pathological mechanisms that determine functional outcomes across lesion locations. The high rate of proptosis resolution in Group II likely results from effective decompression of space-occupying lesions predominating in the cavernous sinus, such as cavernous meningiomas and trigeminal schwannomas, which exert mass effect on orbital structures or obstruct orbital venous outflow. Conversely, the robust improvement of visual acuity in Group I patients may be attributed to the direct alleviation of intraorbital pathology that impinges on the optic nerve or orbital apex.

Variation in functional outcomes and complications across groups suggests that the efficacy of ETOA may be optimized by selecting specific transorbital corridors based on the anatomical target. For example, the vast majority of cavernous sinus lesions are currently accessed via the superior eyelid crease (SLC) ETOA approach (Mathios et al., 2023; Kong et al., 2019; Jeon et al., 2019; Park et al., 2020a, 2020b; Woo et al., 2020; Lee et al., 2021; Lim et al., 2022; Han et al., 2023; Di Somma et al., 2023a, 2023b). However, De Simone et al. recently reviewed the targets and surgical techniques of ETOA, and suggested that switching to the precaruncular (PC) approach may in fact offer a more direct and potentially safer route to the cavernous sinus(De Simone et al., 2024a), concurrently avoiding eyelid incision which may contribute to the high incidence of post-operative ptosis as discussed previously. The current paucity of comparative studies evaluating the efficacy of different ETOA corridors highlights a significant research gap, thus, the suitability of each corridor (e.g., PC, SLC) for distinct anatomical targets remains an attractive target for future investigation.

A recent study by Zoia et al. published the first homogenous series of orbital cavernous haemangioma(Zoia et al., 2025), which falls into Group I (Orbital). The series reported a 25 % rate of transient diplopia, a rate that is similar to our current result. This demonstrates that our anatomical grouping system can provide a helpful framework for risk stratification in surgical approach planning and patient counselling, individualised and tailored to the pathology location. We believe that the anatomically based surgical outcome and risk rate will become more reliable for clinical use with data from future larger-scale studies.

As previously mentioned, we noted that a similar approach to anatomical classification was adopted by Mathios et al., who categorized ETOA-accessible lesions into seven “target zones” (Mathios et al., 2023). However, their study mainly reported aggregate surgical outcomes for the entire cohort without stratifying results by target zone. We believe that our current review bridges the gap between such an anatomical classification system and the elucidation of location-specific risks and functional improvements. We also acknowledge that the inclusion of both lesion epicenter and extension in the classification employed by the Mathios et al. offers a valuable strategy for addressing a key limitation of our study, namely the challenge of classifying lesions that span multiple anatomical regions. Future efforts of systematic analyses could incorporate this epicenter-extension classification system to enhance the rigor of anatomical categorization, potentially guiding surgical planning by identifying optimal ETOA corridors for complex, multi-zone lesions and inform preoperative counselling on location-specific complications.

In view of the potential oversimplification of lesions by anatomical compartments, we have preliminarily explored outcomes by pathology type, with findings summarised in Table 3. Meningiomas, the most frequently reported pathology in our series, were associated with lower rates of gross-total resection (58.8 %) than schwannomas (78.1 %), possibly due to their frequent hyperostosis and multi-compartmental extension that often necessitates planned subtotal removal. Postoperative CSF leak was also more common among the meningioma group (3.3 % vs 0 % for schwannomas), attributable to the need for dural incision and more extensive bone work. In contrast, transient ocular complications were more frequent after schwannoma resection, particularly diplopia (9.4 % vs 7.1 % for meningiomas) and ptosis (15.6 % vs 12.1 % for meningiomas). This is consistent with the predilection of schwannomas for the lateral cavernous sinus and superior orbital fissure, where the cranial nerves for ocular motility traverse. Unfortunately, only 32 schwannomas met our inclusion criteria (compared with >200 meningiomas), which currently limits meaningful statistical comparison between these entities. As more centres adopt ETOA and larger, multicentre datasets become available, we anticipate that further subclassification within each anatomical group, possibly one that incorporates pathology-specific features, will refine prognostic accuracy and better inform preoperative counselling and surgical planning.

**Table 3:** Pathology-specific post-operative outcome and complication rates.

	Meningioma (n = 182)	Schwannoma (n = 32)	Other pathologies (n = 168)
*Gross total resection (GTR)*	58.8 % (107/182)	78.1 % (25/32)	46.4 % (78/168)
***Post-operative improvements***			
*Visual acuity improvement*	64.3 % (54/84)	71.4 % (10/14)	54.5 % (18/33)
*Visual field improvement*	66.7 % (10/15)	100 % (3/3)	40.0 % (2/5)
*Proptosis improvement*	88.6 % (101/114)	90.0 % (9/10)	63.6 % (42/66)
*EOM/diplopia improvement*	60.0 % (18/30)	71.4 % (5/7)	45.5 % (10/22)
***Complications***			
*Postoperative CSF leak*	3.3 % (6/182)	0 % (0/32)	5.4 % (9/168)
*New/worsened diplopia or EOM*	7.1 % (13/182)	9.4 % (3/32)	8.3 % (14/168)
*Postoperative ptosis*	12.1 % (22/182)	15.6 % (5/32)	6.0 % (10/168)
*Other complications*	18.7 % (34/182)	12.5 % (4/32)	17.9 % (30/168)

Lastly, note that we also identified 43 cases which do not contain lesions that fall into the four designated anatomical locations. Most of them consist of traumatic CSF leak repairs and thus fall under the fifth group, “repairs”. The mean age was 42.6 (±15.3). There were 31 males and 12 females. Pathologies are mainly post-traumatic CSF leakage (n = 31), with some cases presenting with concomitant orbital wall fracture requiring decompression (n = 8). All of the remaining cases were encephalocele/meningocele repairs (n = 4) Among the cases with available data, the mean follow-up duration was 10.3 ± 8.4 months. The rate of resolution of CSF leakage post-operatively was 100 %, with no recurrence reported.

### 4.1 Limitations

The results of this study are limited by the inconsistent parameters reported in the original case series. During data collection, we observed a lack of standardized metrics for assessing surgical outcomes and complications across studies, leading to incomplete datasets, limits comparability, and potentially compromise the quality of our analysis. Some assessments were also noted to be rather subjective. For example, cases were reported to have diplopia without supplementary information on the objective assessment of EOM limitation. In addition, some case series documented pre-operative proptosis but failed to state whether there was any improvement, thus severely hindering subsequent analysis. Evidently, no formal meta-analysis was performed because of marked heterogeneity in outcome reporting and follow-up duration. With a high statistical heterogeneity between studies, it lowers the validity of our cross-group chi-square test, our comparisons and p-value analysis remain exploratory rather than inferential. Future meta-analysis with pooled effect estimates can strengthen the statistical validity. Multivariate analysis may also be conducted to independently analyse ETOA surgical outcomes and risks by anatomical site. Therefore, we recommend adopting a standardized set of objective parameters for evaluating surgical outcomes of ETOA in future studies, and, in view of the major parameters related to visual assessment, collaboration with ophthalmologists for formal assessment can potentially be valuable.

In addition, transient cranial nerve deficit can occur after surgery due to tissue manipulation, and possible complete recovery can occur within 3 months of surgery. However, we noticed from our data that some cases did not specify whether the postoperative deficit was transient or persistent, and follow-up durations were occasionally unreported. Providing more detailed information on the postoperative recovery course and temporality of deficits would yield a more comprehensive picture of the surgical outcome of ETOA.

Moreover, we excluded case reports and small series (<5 patients) to ensure statistical reliability, but this likely omitted rare trajectories (e.g., infratemporal or pterygopalatine fossa extensions) and thus led to the inevitable loss of some existing data. The inclusion of predominantly successful series also raises the possibility of publication bias, which may lead to underestimation of true complication rates.

Lastly, the limited number of cases for Group III (extradural) patients, combined with poor outcome reporting in most studies, suggests that the collected data may not fully represent this subgroup's outcomes. As the application of ETOA continues to expand, particularly for extradural lesions such as those in the infratemporal fossa, an increase in reported cases is likely to enhance the availability of comprehensive data in the literature. We envision that this growing body of evidence could facilitate more robust analyses of outcomes not only for Group III patients, but also other anatomical subgroups.

## 5 Conclusions

This systematic review proposed an anatomical grouping system to analyse location-specific surgical risks and outcomes systematically. Our findings again confirm ETOA's efficacy while highlighting the significance of this new classification for anatomy-based risk assessment. A significant limitation identified is the lack of standardised outcome reporting across the current literature. Future research should adopt uniform objective parameters to provide more comprehensive outcomes for this evolving surgical approach.

## Declaration of competing interest

The authors declare that they have no known competing financial interests or personal relationships that could have appeared to influence the work reported in this paper.
